# Pioneer of Medical Mycology: In Memory of Prof. Friedrich Staib, MD, DVM

**DOI:** 10.1111/myc.70203

**Published:** 2026-07-04

**Authors:** Barbara Buchberger, Volker Rickerts

**Affiliations:** ^1^ Museum at the Robert Koch Institute the Public Health Visitor Centre, Robert Koch Institute Berlin Germany; ^2^ Institute for Health Care Management and Research University of Duisburg‐Essen Essen Germany; ^3^ Unit 16 Mycotic and Parasitic Agents and Mycobacteria, Robert Koch Institute Berlin Germany

**Keywords:** 20th century, history, mycology, pathogenic fungus, yeast

## Abstract

At the beginning of Friedrich Staib's academic career in the 1950s, medical mycology was a young discipline in Germany. He studied veterinary and human medicine, earning two doctorates. Initially fascinated by the changes in intestinal flora caused by the newly emerging broad‐spectrum antibacterials, i.e., the selection of fungi. In 1953, he began setting up a mycological laboratory at the University of Würzburg and qualified as a professor in microbiology in 1962. In 1968, he became the head of the mycology department, which he established, at the Robert Koch Institute, Germany's public health Institute in Berlin. His work focused on improving the identification of fungi, both within hosts and the environment. His research on *Cryptococcus neoformans* (Staib agar indicator medium based on the virulence factor melanin for cultivation) gained him international recognition. He was the first to describe secreted proteases from *Candida albicans* as a virulence mechanism of the main human fungal pathogen. Later, nosocomial exposure of immunocompromised individuals to *Aspergillus fumigatus* became an issue for him. He cultivated intensive collaboration with clinicians and pathologists, which had a major influence on his research. Staib's broad interest in fungal diagnostics, virulence factors, and environmental niches of fungal pathogens was groundbreaking for medical mycology in Germany and beyond at a time when systemic mycoses were becoming increasingly important. With his systems thinking, he influenced doctoral students and was a role model for public health.

## 1 Introduction

At the beginning of Friedrich Staib's academic career in the 1950s, medical mycology was a young discipline in Germany. Systemic mycoses in humans were considered extremely rare. Due to improved treatment options for bacterial infections and the introduction of cytotoxic chemotherapeutics and immunosuppressants, systemic mycoses became increasingly important during his lifetime, particularly with the spread of HIV from 1980 onwards [1].

In his work, he achieved technical progress in diagnostic testing for fungal pathogens, novel insights into key questions, including virulence factors, environmental niches, and antifungal therapy.

The growing importance of medical mycology was reflected in the founding of professional societies such as ISHAM in 1953 or the German society DGMyK in 1961, and Staib was actively involved in national and international exchanges. He also established the mycology department at the Robert Koch Institute, Germany's public health Institute in Berlin, in 1968.

Staib has shaped German mycology with his extraordinary passion for research and science, as well as his willingness to take responsibility for human and animal health.

Here, we present a narrative review of the life and achievements of the researcher Friedrich Staib, highlighting his pioneering spirit, collegial attitude, and thorough academic teaching.

## 2 Materials and Methods

We performed interviews with colleagues, doctoral students, and family members to provide insights into the person, the societal, and the times background of Friedrich Staib.

Doctoral theses supervised between 1971 and 1989 and stored at the RKIs archive were screened for research topics and methods (Table S1).

We performed a selective review on published literature concerning key achievements, including secreted proteases of *Candida albicans* and exposure of hospitalised patients to *Aspergillus fumigatus*, focusing on the cultivation and identification of *Cryptococcus neoformans* in human infections and from the environment.

## 3 Results

### 3.1 Upbringing in Tumultuous Times

Friedrich Staib was born on 04 August 1925 in Uhingen (Württemberg) as one of the two sons of master butcher and innkeeper Julius Staib and his wife Emma (Table 1). From May 1943, he was required to perform labour service and was drafted into the German Army (Wehrmacht) shortly after his 18th birthday. Being sent to Russia near the Black Sea, he became a prisoner of the Russian army and was sent to a camp in Odesa. During that difficult situation, he had many life‐changing experiences; yet, as he spoke a bit of Russian, he was able to benefit from his linguistic interest and talent here. After returning home from war, Staib began studying veterinary medicine at Ludwig Maximilian University in Munich in 1945.

**TABLE 1:** Biographical details.

Born on 4 August 1925
Military service and captivity 1943–1945
Studied veterinary medicine in Munich from 1945, and received a doctorate in 1952 (Director of the institute and mentor: Prof. Dr. med. vet. Michael Rolle; formal advisor: Prof. Dr. Anton Meyn) Studied human medicine in Würzburg 1952–1957 Licence to practise 1959 Doctorate 1959 (University of Würzburg, advisor: Prof. Dr. med. Curt Sonnenschein) Habilitation in microbiology 1962 (University of Würzburg, mentor: Prof. Dr. med. Curt Sonnenschein) Professorship at Universität Free University of Berlin 1972
Establishment and management of a mycological laboratory, University of Würzburg 1953–1968
Married Johanna Riedl in 1966
Head of the mycology department at the Robert Koch Institute, 1968–1990
Sons Friedrich born in 1968, Jörg in 1970, Peter in 1971
Died on 18 October 2011 in Sommerhausen

### 3.2 Becoming a Mycologist

During his studies of veterinary medicine, he was trained between 1950 and 1952 at the Institute for Animal Hygiene of the University of Munich in the field of bacteriological examination of human and animal faeces. Using cultivation, he observed shifts in the intestinal flora in favour of yeasts and related this to the increasing use of broad‐spectrum antibiotics, gaining first‐hand insights into what was later termed “Antibiotic‐induced collateral damage” [2].

This new mycological focus fascinated him more than the bacteriological intestinal flora studies practised at the institute at the time, in which he was also involved (personal communication PS). As this shift in intestinal flora was becoming increasingly interesting to human medicine, but the Institute for Animal Hygiene did not yet have any basic knowledge about fungi as pathogens, either from a diagnostic or pathogenetic point of view, collaboration with a competent mycologist was welcomed. This was the biologist and yeast specialist Siegfried Windisch from the Institute of Botany at the University of Munich. He became Staib's first teacher in the field of diagnostics of yeasts and yeast‐like fungi (personal communication, PS).

After a lecture in March 1952 at the Central Clinics in Göppingen, located near his home town Uhingen, on changes in the intestinal flora in favour of fungi after antibiotic therapy, Staib was offered the opportunity to study yeasts in the intestinal tract of patients over a period of about one year in cooperation with the clinic's central laboratory and the gastroenterology department. Windisch, who had since become full professor of microbiology and head of the Department of Microbiology at the Institute for Fermentation Technology in the Department of Agricultural Technology at the Technical University of Berlin, was also recruited as a research partner (personal communication PS). In May 1952, Staib began working in the bacteriological‐serological laboratory at the Central Clinics in Göppingen. The close cooperation between clinicians and laboratory physicians was a formative introduction to human medicine for him (personal communication, PS). To determine the occurrence of yeast species in the stool of inpatients at an internal medicine clinic with a TB ward, the stool of 1158 patients was examined over the course of a year. Yeast fungi of 18 different species were found in 429 (37%) of the 1158 patients [3].

### 3.3 Transition From Veterinary to Academic Research in Human Mycology

It was striking that over 50% of the yeast fungi isolated from inpatients were *Candida albicans* and that certain genera, such as *Cryptococcus*, were absent. One potential reason for this was that there were no differential culture media available for individual yeast genera or species (such as *Cr. neoformans*) to enable accurate identification (personal communication PS). This fact motivated Staib to later work on such media [4, 5, 6].

Curt Sonnenschein, head of the Institute for Hygiene and Microbiology at Julius Maximilian University in Würzburg, also learned about Staib's mycological diagnostic research at the Central Clinics in Göppingen. He offered Staib the opportunity to set up a mycological laboratory while also allowing him to study human medicine (personal communication PS). In the summer semester of 1953, Staib began working as a research assistant at the Institute for Hygiene and Microbiology and as a student of human medicine at the University of Würzburg. As a result of the many subsequent discoveries made here, after passing his medical state examination, Staib was able to complete his doctorate in 1959, and his habilitation already in 1962 [7].

The yeast strains collected and identified in Göppingen and at the Institute for Fermentation in Berlin, along with a large number of cultivated filamentous fungi, served as the basis for the collection to be built up for the mycological laboratory at Würzburg University.

Augusto Chaves Batista from Recife/Pernambuco (Brazil), a renowned biologist and mycologist, had also become aware of Staib and his research. In March 1960, Staib received an invitation to spend several months studying in Brazil, with the generous offer of being able to work freely on his own mycological research questions at medical institutes and clinics at the University of Recife. In return, Batista and his colleagues wanted to gain insight into Staib's ongoing work on the occurrence and significance of yeasts and yeast‐like fungi in the human intestinal tract. Batista also proposed a joint study on yeast‐like and filamentous fungi from cattle faeces [8]. Staib received funding from the German Research Foundation for his study visit to Brazil, a country harbouring among the world's largest fungal diversity, and he made such an impression in Recife that a fungus was named after him (*Staibia connari, MycoBank 5181*) [9, 10].

### 3.4 Discovery of Melanin Production for Identification of *Cryptococcus neoformans*

The first human isolate of *Cryptococcus* was obtained in 1894 by Otto Busse from the Institute of Pathology and Abraham Buschke from the Clinic for Surgery at the University of Greifswald from a sarcoma‐like lesion in the tibia of a 31‐year‐old woman who later died of disseminated infection [11]. Both suspected a *Coccidium* species, and Friedrich Löffler confirmed its mycotic nature [11]. Later that year, Francesco Sanfelice reported on a similar yeast that he had isolated from fermented peach juice and named *Saccharomyces neoformans* due to its colony form. Jean‐Paul Vuillemin renamed the pathogen *Cryptococcus neoformans* in 1901, as it does not exhibit the characteristic feature of *Saccharomyces*, namely the formation of ascospores [12].

After *C. neoformans* was first isolated from pigeon droppings in 1955 [13], Staib provided cultural evidence for bird droppings as a habitat of *Cr. neoformans* by cultivation from cage sand and droppings of cage birds in 1961 [7, 14]. Using powdered canary droppings, he observed an intense brown colouration of *Cr. neoformans* colonies grown on an agar containing melanin precursors [5]. The examination of droppings from various bird species under the same conditions showed that goldfinches and bullfinches also harboured *C. neoformans* , in contrast to pigeons, budgerigars, and cormorants. Sensitivity analyses of the plant seeds in feed mixtures for these bird species showed that only components of the fruit of *Guizotia abyssinica* produced the brown colouration. Therefore, this seed became an ingredient of the so‐called Staib agar, which is still widespread for the cultivation and identification of human pathogenic, melanin‐producing *Cryptococcus* species [5].

Staib reported on this discovery of melanin as a virulence factor of *Cr. neoformans* at the annual meeting of the International Society for Human and Animal Mycology (ISHAM) during the eighth international congress for microbiology in Montreal in August 1962 [15]. The ISHAM had been founded a few years earlier in Rome in 1954, and one of the hosts, Pietro Redaelli, became its first president. Raymond Vanbreuseghem was appointed as the first secretary general, and Heinz Seeliger, who later succeeded Curt Sonnenschein in Würzburg, was also one of the founding members [16].

After Staib et al. succeeded in developing an indicator medium for isolating *C. neoformans* that contained components of the fruit of *Guizotia abyssinica* , they presented their findings to the Société Française de Microbiologie in Paris on 2 December 1965 [17]. In the publication, the authors note in a footnote that while editing the article, they learned that their colleagues Alston B. Shields and Libero Ajello had submitted an article to the journal Science on 29 November 1965 describing an almost identical formula for the selective medium [17, 18]. They stressed that the research groups had arrived at their results independently of each other and gave priority to the results of their American colleagues [17]. The latter, in turn, referenced Staib's early publications on the subject, as well as his lecture at ISHAM in Canada in 1962. Staib's discovery of the uniqueness of melanin formation for the species *Cr. neoformans* was significant, which is why the culture medium is now known worldwide as Staib agar.

Thirty years later, Polacheck emphasised the far‐reaching and multidisciplinary implications of Staib's discovery and its further development through the combination of Staib agar with membrane filtration technology for the detection of *C. neoformans* in the urine of AIDS patients [19, 20].

**FIGURE 1:**
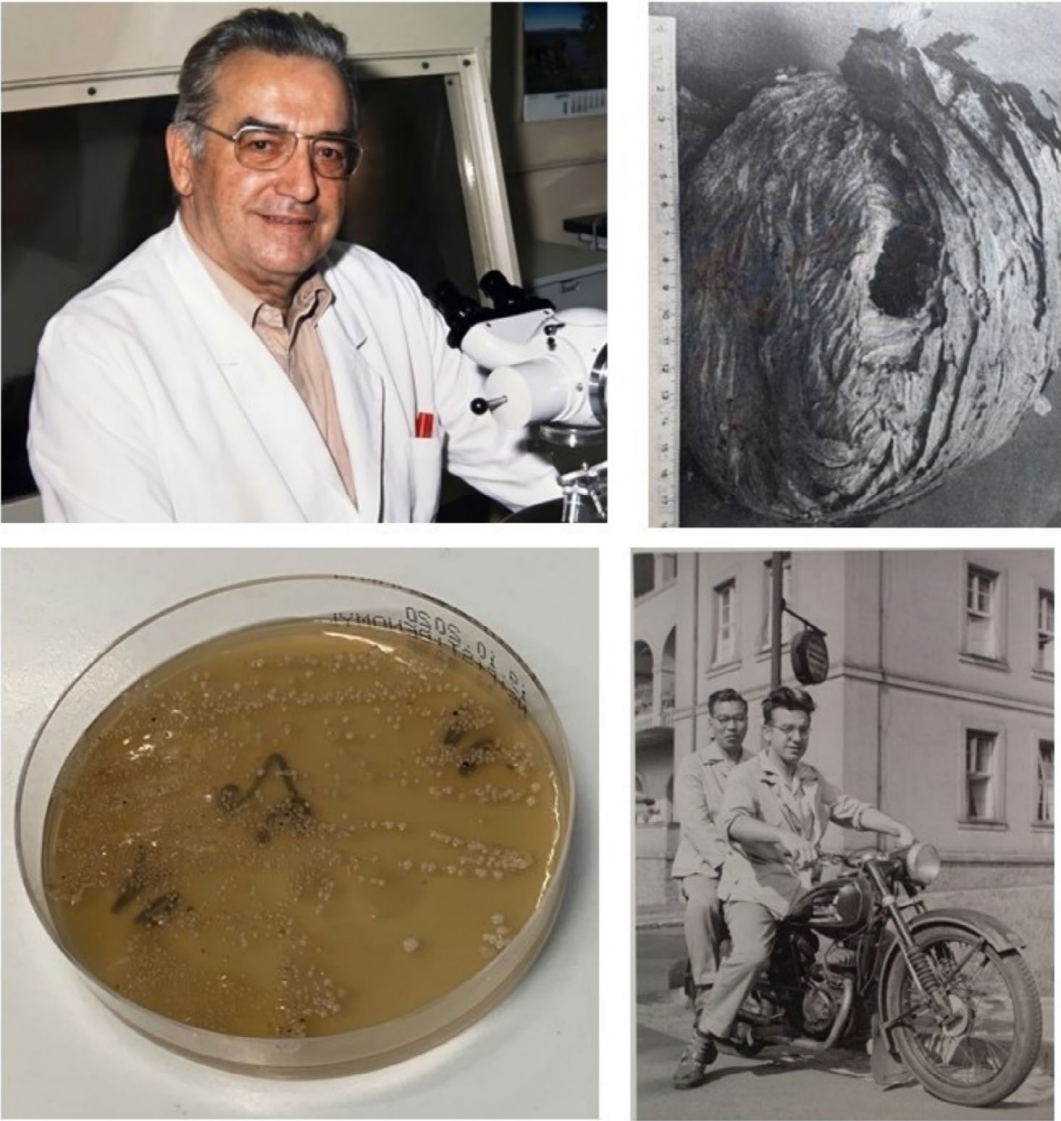
Top left: Friedrich Staib at the laboratory, Berlin, circa 1975, source: TA. Top right: Based on the association of *C. neoformans* with fruits, insects became a study object in search of habitats of this pathogenic fungus. In a dissertation, 120 inactive nests of wasps were cultivated. Yeasts grown were identified, including the so‐called brown colour effect of *C. neoformans* on Staib's Agar from Grave B. Ueber die Suche nach *Cryptococcus neoformans* im Wespennest (Dolichovespula saxonica). Ein Beitrag zur Ökologie und Epidemiologie von Arten der Gattung Cryptococcus (Table S1). Bottom left: Staib's agar, here in a modification, is still the standard approach to cultivate *C. neoformans* from the environment or animal mucous membranes (Koala), where the fungus might be a minority fungal population difficult to identify without indicator media. Bottom right: Staib with Japanese medical mycologist Saneshige Ata, a life‐long friendship, Würzburg in the 1950s, source: FS, PS.

The Agar continues to be used in diagnostic mycology. In addition, by adding the antifungal Benomyl, the agar's performance to identify environmental niches of *C. neoformans* and *C. gattii* was improved by inhibition of rapidly growing moulds but preserving the brown colour effect of the agents of cryptococcosis (Figure 1) [21]. The use of this agar enabled prospective, multi‐researcher efforts to cultivate Cryptococcus from the environment in Europe and the Mediterranean area conducted by the ISHAM Cryptococcus typing group (https://www.isham.org/working‐groups/cryptococcus/) [22]. This research demonstrated a high genetic diversity of environmental isolates and the predominance of a limited number of worldwide distributed sequence types, raising questions on virulence. In addition, this provided information on environmental factors associated with the cultivation of *C. neoformans* and the emerging *C. gattii* in Europe [23].

### 3.5 Virulence of the Predominant Human Yeast Pathogen *Candida albicans*

Another pioneering achievement by Staib was the discovery of proteolytic activity as a principle of pathogenicity in *Candida albicans* and the finding that the breakdown of serum proteins is not species‐specific but strain‐specific for *C. albicans* [24]. In animal experiments, Staib was able to confirm the results through corresponding pathological‐anatomical changes and, based on this, advance research into the human pathogenicity of *C. albicans* [25].

When Staib became the first German to receive the ISHAM Lucille K. George Award in Adelaide in 1994, the president of the society and expert on *C. albicans* , Frank Odds, said in his laudatory speech: ‘Staib is undoubtedly best known for his pioneering work with proteolysis in *Candida albicans* , which opened up the very busy field of study of *Candida* proteinase as a virulence factor and a positive diagnostic tool’ [26].

### 3.6 Establishment of Medical Mycology at the Robert Koch Institute

In 1968, Staib followed an appointment as Director and Professor at the Robert Koch Institute (RKI) in Berlin, which was followed by his transfer of habilitation to the Free University (FU) of Berlin. Staib welcomed this important step in his career, also since Seeliger wanted him to shift the focus of research and diagnosis in Würzburg to Toxoplasmosis. Georg Henneberg, then director of the RKI, had recognised the need to establish a department for medical mycology. Staib became head of the central diagnostic laboratory and also head of the newly founded department for mycology [27]. Due to the lack of university and government institutions for medical mycology at the time, the new department at the RKI also became an institution for research, teaching, and further education [27]. Junior researchers were instructed on research projects including environmental niches of Cryptococcus associated with plants, insects and animals, in vitro resistance testing of antifungals and their effectiveness in animal models, the epidemiology of dermatophyte infections, epidemiologic studies on candidiasis and pathogenicity of sporotrichosis (Table S1).

Staib valued laboratory work, in particular, crucial contributions by technicians who were featured as Coauthors in research articles (personal communication, TA).

In Berlin, Staib continued fruitful research collaborations with clinicians, in particular with pathologist Gernot Grosse, infectious diseases physician Hans Dieter Pohle, and dermatologist Günter Stüttgen (personal communication, TA). The latter was honoured by the USA for his extraordinarily courageous efforts in the Battle of Hürtgen Forest in November 1944, when he negotiated with the enemy to allow each side to recover their dead and wounded during agreed ceasefires [28]. At that time, German and American medical soldiers worked together to care for the wounded and transport them away.

In his search for environmental niches, particularly for *C. neoformans* , Staib repeatedly undertook excursions to greenhouses, the zoo, the underground, Berlin's then‐luxury department store KaDeWe, and pigeon breeders. Samples were taken everywhere to cultivate *C. neoformans* on Staib's agar to find environmental niches of this fungus and mechanisms of distribution in the environment (personal communication, TA, Figure 1).

At the Berlin Heart Centre, he also examined the potting soil of indoor plants and identified it as a biotope for facultative pathogenic filamentous fungi, *Aspergillus fumigatus*, among them [29]. In an aim to protect immunocompromised patients from environmentally acquired mould infections, he was so energetic that he was temporarily banned from the hospital, yet followed by fruitful collaboration (personal communication, TA, FS, PS).

Even after his retirement in 1990, Staib remained tirelessly active in scientific research, focusing,e.g., on the potential medical and mycological effects of the introduction of organic waste bins in the mid‐1990s [30].

### 3.7 Highly Valued Mycologist in Berlin and Beyond

‘You have to get along with everyone, you have to be a diplomat,’ was Staib's motto in life. However, when human lives were at risk, he took a stand and acted decisively. Staib was very approachable and helpful; he responded immediately to short‐notice requests but was also able to immerse himself deeply in research (personal communication, TA). He was an outstanding diagnostician for fungal infections, and his advice was highly valued, especially by clinicians with chronically ill lung and haematological‐oncological patients, but also by pathologists [31].

As a university lecturer, he was very patient and conscientiously supervised a sizeable collection of doctoral theses (Table S1); he taught his students to think in systems (personal communication, TA).

Staib was particularly keen to maintain contact with mycologists in the former German Democratic Republic (GDR) and the Eastern Bloc countries. The fact that he had learned Russian in his youth facilitated communication. Every encounter with West German colleagues was something special because otherwise they would not have learned about many developments in Medical Mycology (personal communication, CS).

Like Robert Koch, who was friends with the Japanese physician and bacteriologist Shibasaburo Kitasato, Staib was friends with the Japanese medical mycologist Saneshige Ata, whom he met in Würzburg. Also, like Robert Koch, Staib was granted an audience with the Japanese emperor when the ISHAM annual conference was held in Tokyo in 1975. On this occasion, he performed two Japanese songs and impressed the emperor (personal communication FS, PS).

### 3.8 Family and the Sommerhausen Refuge

Another passion of Staib's, alongside mycology, was renovating his historic house. He had purchased a Baroque‐era pharmacy in Sommerhausen near Würzburg, and this Franconian wine village was his refuge and, at times, a place of longing. During the summer holidays, he enjoyed rummaging through old farmhouses and barns to discover building materials that he could use in the restoration of the pharmacy (personal communication, TA, FS, PS). Staib's passions were also passed on to his sons: Friedrich became an architect with expertise in the preservation and restoration of historic buildings, Peter became a mycologist, and Joerg an engineer; all three now live in historic buildings (personal communication, TA, FS, PS). The central figure in the family was Staib's wife, Johanna. She organised family life and accompanied her husband on trips that took him to conferences, and research stays in Brazil, Holland, Belgium, Canada, England, the USA, France, India, Japan, Israel, Russia and various Eastern Bloc countries (personal communication, TA, FS, PS) [31]. After Staib's retirement, she also took over the organisation of his lectures and publications.

### 3.9 Awards and Conclusion

Staib has published his research in over 240 journal articles and book contributions and has gained worldwide recognition. For outstanding achievements in the field of microbiology, he was awarded the Aronson Prize by the Berlin Senate in 1976, honorary membership of the ISHAM in 1994, in 1998 he was made an honorary member of the German‐speaking Mycological Society (DMykG), and in 2005 he was awarded the Schönlein medal by the DMykG in recognition of his achievements.

Staib's broad interests, ranging from fungal diagnostics and fungal virulence factors to the environmental niches of fungal pathogens, were groundbreaking for medical mycology in Germany at a time when systemic mycoses were becoming increasingly important and remain an inspiration today.

Always driven by the search for the cause of a problem, his panoramic view also helped him on excursions to find potential sources, such as at pigeon breeders or food departments.

In addition to thinking in terms of systems, he found inspiration for his research in the conscious and frequently sought exchange with clinical colleagues.

In his obituary for Staib, Claus Seebacher writes: ‘His significant scientific life's work remains as a foundation for the memory of an extremely remarkable and amiable personality’ [31].

## Author Contributions

**Volker Rickerts:** writing – review and editing, supervision. **Barbara Buchberger:** conceptualization, writing – original draft.

## Conflicts of Interest

The authors declare no conflicts of interest.

## Supporting information


**Table S1:** PhD theses of Prof. Dr. med. Dr. med. vet. Friedrich Staib's doctoral students at the Free University of Berlin available at the Robert Koch Institute's library.

## Data Availability

Data sharing not applicable to this article as no datasets were generated or analysed during the current study.
