# Supplementary material for: Pioneer of Medical Mycology: In Memory of Prof. Friedrich Staib, MD, DVM
Source: Mycoses. 2026 Jul 4;69(7):e70203. doi: 10.1111/myc.70203 (PMC13332417; doi:10.1111/myc.70203)
Supplement: Supplementary file 1 — Table S1: PhD theses of Prof. Dr. med. Dr. med. vet. Friedrich Staib's doctoral students at the Free University of Berlin available at the Robert Koch Institute's library. [file MYC-69-e70203-s001.docx]

Supplementary Table 1: PhD theses of Prof. Friedrich Staib’s doctoral students at Free University of Berlin available at the Robert Koch Institute’s library

| **Year** | **PhD Candidate** | **Title of thesis** | **Link to DNB** |
| --- | --- | --- | --- |
| 1971 | Reinhild Geier | Zum Wachstum von Staphylococcus aureus im Proteolyse-Kulturfiltrat von Candida albicans | https://d-nb.info/720386322 |
| 1972 | Hans-Joachim Haßemer | Mykologisch-epidemiologische Untersuchungen über eine Mikrosporie-Endemie in Uganda | https://d-nb.info/740940953 |
| 1972 | Gernot Grosse | Zur experimentellen Sporotrichose der Maus: Pathologisch-Anatomische Studie der applikationsabhängigen Reaktion des Organismus | https://d-nb.info/730627861 |
| 1973 | Gudrun Altmann (geb. Grühn) | Über die Proteolyse bei Candida albicans und die antimyzetische Wirkung von Nystatin | https://d-nb.info/751102598 |
| 1975 | Wolfgang Preuss Erhard Radlmeier | Das Cryptococcom und Amphotericin B: Tierexperimentelle Untersuchungen zur Therapie der Cryptococcose | https://d-nb.info/760687560 |
| 1975 | Bernhard Grave | Über die Suche nach Cryptococcus neoformans im Wespennest (Dolichovespula Saxonica) : Ein Beitrag zur Ökologie und Epidemiologie von Arten der Gattung Cryptococcus | https://d-nb.info/760670552 |
| 1976 | Lutz Altmann | Über die Suche nach Cryptococcus neoformans an Pflanzensamen: Ein Beitrag zur Ökologie und Epidemiologie von Arten der Gattung Cryptococcus | https://d-nb.info/770834760 |
| 1977 | Thomas Abel | Systemische Sprosspilzmykosen und ihre Therapie, Sprosspilze in Berlin und ihre Emfindlichkeit gegen 5-Fluorcytosin | https://d-nb.info/780701119 |
| 1979 | Martin Focking | Über den Candida-albicans-Nachweis bei Blutspendern : Ein Beitrag zur Diagnostik und Pathogenese der Candida- Albicans-Mykose | https://d-nb.info/801286506 |
| 1979 | Regina Berger Schoon, Anne | Über das Vorkommen von Sprosspilzen im Sektionsgut | https://d-nb.info/790961121 |
| 1980 | Ingeborg Lampe (geb. Weber) | Über die Rotpigment-, und Chlamydosporenbildung bei Trichophyton rubrum | https://d-nb.info/810014475 |
| 1989 | Maria Heißenhuber | Vogelfäkalien als Cryptococcus-neoformans-Standorte in Berlin (West): Ein Beitrag zur Bekämpfung der Cr.-Neoformans-Infektion | https://d-nb.info/891477241 |
| DNB: German National Library (Deutsche Nationalbibliothek) | | | |
